# Ficolin-A induces macrophage polarization to a novel pro-inflammatory phenotype distinct from classical M1

**DOI:** 10.1186/s12964-024-01571-4

**Published:** 2024-05-15

**Authors:** Li-Wen Zhu, Zihao Li, Xiaohong Dong, Huadong Wu, Yifan Cheng, Shengnan Xia, Xinyu Bao, Yun Xu, Runjing Cao

**Affiliations:** 1 Center for Rehabilitation Medicine, Department of Neurology, Zhejiang Provincial People’s Hospital, Affiliated People’s Hospital, Hangzhou Medical College, Hangzhou, Zhejiang China; 2 Department of Neurology, Nanjing Drum Tower Hospital Clinical College of Nanjing University of Chinese Medicine, Nanjing, Jiangsu China; 3 Department of Neurology, Nanjing Drum Tower Hospital, Medical school of Nanjing University, Nanjing, Jiangsu China; 4 Department of Neurology, Shaoxing People’s Hospital, Shaoxing, China; 5 The Affiliated Lianyungang Hospital of Xuzhou Medical University, The First People’s Hospital of Lianyungang, Lianyungang, Jiangsu China

**Keywords:** Ficolin-A, Macrophage, Phenotype, Autoimmune disease

## Abstract

**Background:**

Macrophages are key inflammatory immune cells that orchestrate the initiation and progression of autoimmune diseases. The characters of macrophage in diseases are determined by its phenotype in response to the local microenvironment. Ficolins have been confirmed as crucial contributors to autoimmune diseases, with Ficolin-2 being particularly elevated in patients with autoimmune diseases. However, whether Ficolin-A stimulates macrophage polarization is still poorly understood.

**Methods:**

We investigated the transcriptomic expression profile of murine bone marrow-derived macrophages (BMDMs) stimulated with Ficolin-A using RNA-sequencing. To further confirm a distinct phenotype activated by Ficolin-A, quantitative RT-PCR and Luminex assay were performed in this study. Additionally, we assessed the activation of underlying cell signaling pathways triggered by Ficolin-A. Finally, the impact of Ficolin-A on macrophages were investigated in vivo through building Collagen-induced arthritis (CIA) and Dextran Sulfate Sodium Salt (DSS)-induced colitis mouse models with Fcna-/- mice.

**Results:**

Ficolin-A activated macrophages into a pro-inflammatory phenotype distinct to LPS-, IFN-γ- and IFN-γ + LPS-induced phenotypes. The transcriptomic profile induced by Ficolin-A was primarily characterized by upregulation of interleukins, chemokines, iNOS, and Arginase 1, along with downregulation of CD86 and CD206, setting it apart from the M1 and M2 phenotypes. The activation effect of Ficolin-A on macrophages deteriorated the symptoms of CIA and DSS mouse models, and the deletion of Fcna significantly alleviated the severity of diseases in mice.

**Conclusion:**

Our work used transcriptomic analysis by RNA-Seq to investigate the impact of Ficolin-A on macrophage polarization. Our findings demonstrate that Ficolin-A induces a novel pro-inflammatory phenotype distinct to the phenotypes activated by LPS, IFN-γ and IFN-γ + LPS on macrophages.

**Supplementary Information:**

The online version contains supplementary material available at 10.1186/s12964-024-01571-4.

## Introduction

Macrophages are the primary immune cells widely distributed in tissues, playing an important role in inflammation, cell death, and homeostasis maintenance [1, 2]. They are characterized with high plasticity in response to various microenvironmental stimuli, resulting in polarization into distinct macrophage phenotypes [3]. The two major phenotypes recognized are classically activated/inflammatory (M1) and alternatively activated/anti-inflammatory (M2) macrophages. M1 phenotype macrophages mainly express pro-inflammatory mediators including IL-1, IL-6, reactive nitrogen and oxygen intermediates, which contribute to microbicidal and tumoricidal activities. Conversely, M2 phenotype macrophages express anti-inflammatory molecules like Arginase-1, Mrc1(also known as CD206), IL-10, involved in parasite infestation, tissue remodeling [4]. However, macrophages are activated to a spectrum rather than clearly defined M1 and M2 phenotypes in stimulation of various molecules. In certain diseases, macrophages are activated and shifted to a phenotype which results in dysregulated and persistent inflammation and exacerbate the pathophysiological process [5]. Therefore, exploring molecules regulating macrophage biology and polarization is critical to effectively interfering the progression of diseases.

Ficolins are a group of proteins that were initially discovered on porcine uterus membranes in 1993 as a transforming growth factor (TGF)-b1-binding protein [6]. Ficolins have been confirmed to play a role in triggering the complement lectin pathway and protecting host against invading pathogens. Ficolins are identified as innate soluble pattern recognition molecules (PRMs) that form complexes with mannose-binding lectin-associated serine proteases (MASPs) [7]. Several members of the ficolin family have been identified in human and mouse so far, including human M-ficolin (ficolin-1 or FCN1), L-ficolin (ficolin-2 or FCN2), human H-ficolin (ficolin-3 or FCN3), mouse ficolin-A (FCNA), and mouse ficolin-B (FCNB)) [8, 9]. Mouse FCNA resembles human FCN2 based on the structural and functional properties. FCNA mainly produced and secreted by hepatocytes and present in blood as serum lectins. It has a lectin-like activity by binding to 1, 3-β-D glucan (GlcNAc), which can be find on the cell walls of yeast and fungal [10, 11].

Emerging evidence suggests that FCN2 plays a significant role in the onset and progression of autoimmune diseases. Previous study has reported an elevated serum FCN2 concentration in patients with Rheumatoid arthritis (RA), where the elevated serum FCN2 level showed positive correlation with swollen joint counts (SJCs) and rheumatoid factor (RF) [12]. Similarly, higher titers of FCN2 were also found in Crohn’s disease (CD), a form of inflammatory bowel disease (IBD) [13, 14]. While higher titers of FCN2 indicates its role in autoimmune diseases, more in-depth studies are necessary to elucidate the underlying mechanisms.

Macrophage polarization has great importance in the progression and prognosis of autoimmune diseases. Current treatments for autoimmune diseases, including RA and IBD, focus on the regulating and shifting the polarization of macrophages [15–17]. Previous reports have highlighted the relevance and critical role of Ficolins in autoimmune diseases [13, 18]. What’s more, FCN2 was proved to defend against virulent M. tuberculosis H37Rv infection by stimulating macrophages to release IL-6, TNF-α and nitric oxide (NO) [19]. However, whether FCN2/A activates macrophage polarization into a pro-inflammatory phenotype haven’t been clarified. Therefore, we opted to map the transcriptome profile by RNA sequencing (RNA-Seq) to further define the polarization pattern induced by FCNA, which might be a therapeutic target for autoimmune diseases. Our results indicate that FCNA induces a distinct transcriptional program and promotes macrophage polarization into a distinct phenotype.

## Materials and methods

### Mice

C57BL/6J mice (8 weeks old) and DBA/1 mice (8 weeks old) were purchased from Nanjing Biomedical Research Institute of Nanjing University. Fcna^−/−^ mice on a C57BL/6 N background were generated by Cyagen Biosciences (Nanjing, China). All mice were maintained at the Nanjing Drum Tower Hospital Animal Center under specific-pathogen-free conditions. Age- and sex-matched mice were used for all experiments. Fcna^−/−^ mice and their wild type (WT) littermates were used for the induction of CIA and DSS-induced colitis. All animal experiments were conducted according to the protocol approved by the Institutional Animal Care and Use Committee of Nanjing Drum Tower Hospital.

### Cell culture and stimulation

Bone marrow-derived macrophages (BMDMs) were prepared as previously described [20]. Briefly, on the first day, 8-week-old C57BL/6J mice were sacrificed and sterilized with 70% ethanol. The femur and tibia were isolated, and all the muscle tissue was removed from the bones. The bone marrow was flushed out using lymphocyte medium and pipetted thoroughly to create a single-cell suspension. The cells were then passed through a cell strainer and adjusted to a concentration of 2 × 10^6 cells/ml in lymphocyte medium containing 10% L929-conditioned medium (BMDMs medium). The cells were then plated in a 15-cm culture dish and differentiated in a humidified incubator at 37℃ with 5% CO2 for 7 days.

For RNA-sequencing, BMDMs pretreated with recombinant mouse IFN-γ (R&D) (20 ng/ml) for 20 h followed by Lipopolysaccharide (LPS, Sigma-Aldrich) (500 ng/ml) stimulation for 4 h were referred to ‘IFN-γ + LPS’ group; BMDMs treated with IFN-γ (20 ng/ml) for 24 h were referred to ‘IFN-γ’ group; BMDMs treated with recombinant mouse FCNA (CUSABIO) (500 ng/ml) for 24 h were referred to ‘FCNA’ group. For quantitative RT-PCR, BMDMs were treated with none, 200 ng/ml FCNA, and 500 ng/ml FCNA respectively. For western blotting, BMDMs were treated with 500 ng/ml FCNA for 0, 15, 30, 45, and 60 minutes respectively. BMDMs treated with FCNA (500 ng/ml) followed by SB202190 (50nM), Ruxolitinib (5µM), PDTC (10µM), SCH772984 (300nM), SP600125 (10nM) respectively were subjected to quantitative RT-PCR. Supernatants of BMDMs treated with or without FCNA (500 ng/ml) were collected to be used as conditioned medium for MODE-K cells.

The murine enterocyte cell line, MODE-K, was purchased from BLUEFBIO™ (Shanghai, China) and cultured in Dulbecco’s Modified Eagle Medium (DMEM, Invitrogen) supplemented with 10% Fetal bovine serum, 100 IU penicillin, and 100 mg/ml streptomycin in a humidified incubator at 37℃ with 5% CO2. For cell apoptosis detection and cell viability detection, MODE-K cells were cultured with half general medium and half conditioned medium from Control or FCNA-treated BMDMs. For LPS stimulation, MODE-K cells were incubated with 500ng/ml LPS for 24 h.

### Flow cytometry detecting cell apoptosis

MODE-K cells were digested with Trypsin (Beyotime) without ethylenediaminetetraacetic acid (EDTA) at room temperature for 1 min. The digestion was terminated by adding DMEM containing 10% FBS. The cells were centrifuged at 2000 rpm at 4 ℃ for 5 min and the supernatant was removed. The cells were washed twice with pre-cooled 1 × PBS and resuspended in 1× Annexin V binding buffer. According to the protocols of the Annexin V-PE/7-AAD Apoptosis Detection Kit (Vazyme), cells were stained with 5 µl Annexin V-PE and 5 µl 7-AAD staining solution and examined by flow cytometry (LSRFortessa™, BD Biosciences). The apoptosis rate is the sum of early apoptosis (Annexin V positive/7-AAD negative) and late apoptosis (Annexin V positive/ 7-AAD positive).

### CCK-8 assay detecting cell viability

MODE-K cell viability was detected by Cell Counting Kit-8 (CCK-8, Beyotime). Cells were seeded in a 96-well plate for 24 h following different treatments. The cells were then added with 10 µl of CCK-8 solution, and incubated in an incubator at 37 °C for 2 h. The absorbance at 450 nm (OD450) was detected by a microplate reader (Bio-Rad).

### Reagents

The reagents and antibodies used in this study were listed in Table S2.

### RNA sequencing (RNA-seq)

Total RNA of BMDM cells were extracted using TRIzol reagent (Invitrogen) according to the manufacturer’s instructions, and genomic DNA was removed by DNase I (TaKara). The RNA quality was determined by 2100 Bioanalyser (Agilent) and quantified using the ND-2000 (NanoDrop Technologies). High-quality RNA samples (OD260/280 = 1.8 ∼ 2.2, OD260/230 ≥ 2.0, RIN ≥ 6.5, 28 S:18 S ≥ 1.0, > 1 µg) were used to construct sequencing library. After further purification, the RNA samples were used for library construction. Paired-end RNA-seq sequencing library was sequenced with the Illumina HiSeq xten/NovaSeq 600 sequencer (2 × 150 bp read length). The differential expression analysis was performed using the edgeR ( http://www.bioconductor.org/packages/2.12/bioc/html/edgeR.html )with Q value ≤ 0.05. Differentially expressed genes (DEGs) with |log2FC|>1 and Q value ≤ 0.05 were considered to be significant DEGs. The Kyoto Encyclopaedia of Genes and Genomes (KEGG) enrichment analysis was carried out by KOBAS [21] ( http://kobas.cbi.pku.edu.cn/home.do ).

### Luminex

The concentration of IL-1α, IL-1β, IL-2, IL-3, IL-4, IL-5, IL-6, IL-9, IL-10, IL-12 p40, IL-12 p70, IL-13, IL-17 A, IFN-γ, CXCL1, CCL2, CCL5, G-CSF, GM-CSF, Eotaxin, and TNF-α in serum were quantified with the Luminex 200^™^ (Lumine Xmap Technology) coupled with xPONENT software according to manufacturer’s instructions. In brief, supernatants of BMDM cells treated with or without FCNA were collected and incubated in 96-well plates embedded with microbeads for 30 min, and then incubated with detection antibodies for 30 min. Finally, streptavidin-PE was added into each well for 10 min, and values were read using the BioPlex MAGPIX System (Bio-Rad).

### Western blotting

Cells were washed with pre-cooled PBS and lysed on ice for 30 min with pre-cooled RIPA lysis buffer containing a cocktail of protease inhibitors and phosphatase inhibitors. Cellular debris was clarified (12,000 rpm, 30 min, 4℃). Cell lysate was prepared and subjected to western blotting as previously described [22]. The membranes were incubated with the following primary antibodies (1:1000) on a shaker at 4℃ overnight: p-NFκB, NFκB, p-IκB, IκB, p-P38, P38, p-ERK, ERK, p-JNK, JNK, p-JAK2, JAK2, p-STAT1 S727, p-STAT1 T701, STAT1, and β-actin (Table S2). After washed with 0.1% Tris-buffered saline with Tween 20 (TBST) for 3 times (10 min each), the membranes were incubated with HRP-labeled secondary antibodies (1:5000) for 2 h at room temperature. The color was detected with an ECL Western Blotting Detection kit (Millipore).

### RNA isolation and quantitative RT-PCR

Total RNA of BMDM cells were extracted using TRIzol reagent (Invitrogen) according to the manufacturer’s instructions. Reverse transcription was conducted using qPCR RT Master Mix (Vazyme, Nanjing, China). Real-time PCR was carried out using SYBR Green Master Mix (Vanzyme, Nanjing, China) and the LightCycler 96 instrument (Rouche). The mRNA expression level was normalized and determined using the 2-ΔΔCt method. Primer sequences are shown in Table S1.

### Induction and evaluation of collagen-induced arthritis (CIA)

8-week-old DBA/1 mice and Fcna^−/−^ and WT littermate mice were used to induce CIA. The CIA modeling procedure was described in previous research [23]. Immunization grade bovine type II collagen (CII) was emulsified with complete Freund’s adjuvant (CFA) on ice at a ratio of 1:1. The emulsion was injected intradermally 1.5 cm distal from the base of the tail. A booster immunization was administrated on the 21st day, following the same method as the primary immunization. Redness and swelling of joints in the limbs of mice represent the successful modeling of CIA. The evaluation of CIA severity is based on scoring for arthritis severity according to previous work [23]. The scoring schemes for Histological synovitis score (HSS) and Osteoarthritis Research Society International (OARSI) score are based on the previous work (Table S3-S4) [24].

### Induction and evaluation of DSS-induced colitis

8-week-old Fcna^−/−^ mice and WT littermates were used to induce colitis by replacing their drinking water with a 2% (wt/v) DSS (M.W. = 36,000–50,000 Da, MP Biomedicals) solution for 8 days. The body weight and general condition of mice were observed daily during the experiment. On day 8, mice were sacrificed and colons were collected for length measuring and H&E staining. The histological scoring scheme was based on the previous work (Table S5) [25].

### Safranine O fast green staining

All the experimental mice used to induce CIA were sacrificed, and ankle joints were obtained at 42 days after the primary immunization. The ankle joints were fixed with 4% paraformaldehyde for 72 h, and decalcified with EDTA decalcification solution for 8 weeks, with the solution changed every 4 days. Remove the decalcification solution until the syringe needle could easily pierce into the tissue. Subsequently, the tissues were dehydrated, made transparent, waxed, and embedded in paraffin. 4 μm paraffin sections were prepared from sagittal view with a paraffin microtome (Leica, Wetzlar, Hessen, Germany), and stained with Modified Safranine O-Fast Green FCF Cartilage Stain Kit (Solarbio, Wuhan, China). The histopathological alterations were captured by microscope (IX73, Olympus, Tokyo, Japan).

### Toluidine blue staining

The ankle joints were decalcified, embedded in paraffin wax and sliced as previously described. The slices were dewaxed with xylene and gradient ethanol. The sections were then stained with toluidine blue for 30 min, washed with tap water, and dehydrated in grade ethanol. Xylene was dropped on the slices to make the tissues transparent, and rhamsan gum was finally added for sealing. The histopathological alterations were captured by microscope (IX73, Olympus, Tokyo, Japan).

### Hematoxylin-Eosin staining and histological scores

The ankle joints of CIA mice were decalcified, embedded in paraffin wax and sliced as previously described. For mice with DSS-induced Colitis, colons were cut along the longitude axis and washed with normal saline. The colon tissues were then fixed with 4% paraformaldehyde overnight, embedded with paraffin wax, and sliced as before. The sections were stained with hematoxylin and Eosin (H&E). The histopathological alterations were observed and captured by microscope (IX73, Olympus, Tokyo, Japan).

### Statistical analysis

GraphPad Prism 9.0 was used for all data analysis. The data were presented as the mean values ± SEM. The statistical significance of differences in mean values were determined by unpaired Student’s t test for two group analysis, and two-way ANOVA for multiple group analysis. P values less than 0.05 were considered statistically significant (* *P* < 0.05, ** *P* < 0.01, *** *P* < 0.001, **** *P* < 0.0001).

The sample size for each experiment was calculated using the program G*Power version 3.1.9.7 (Faculty of Mathematics and Natural Sciences, Dusseldorf, Germany) considering the power of 0.80 and alpha error probability of 0.05 for all tests and taking into account the drop rate of experimental animals of 10%.

## Results

### FCNA induces a distinct transcriptional program in macrophages

**Fig. 1:**
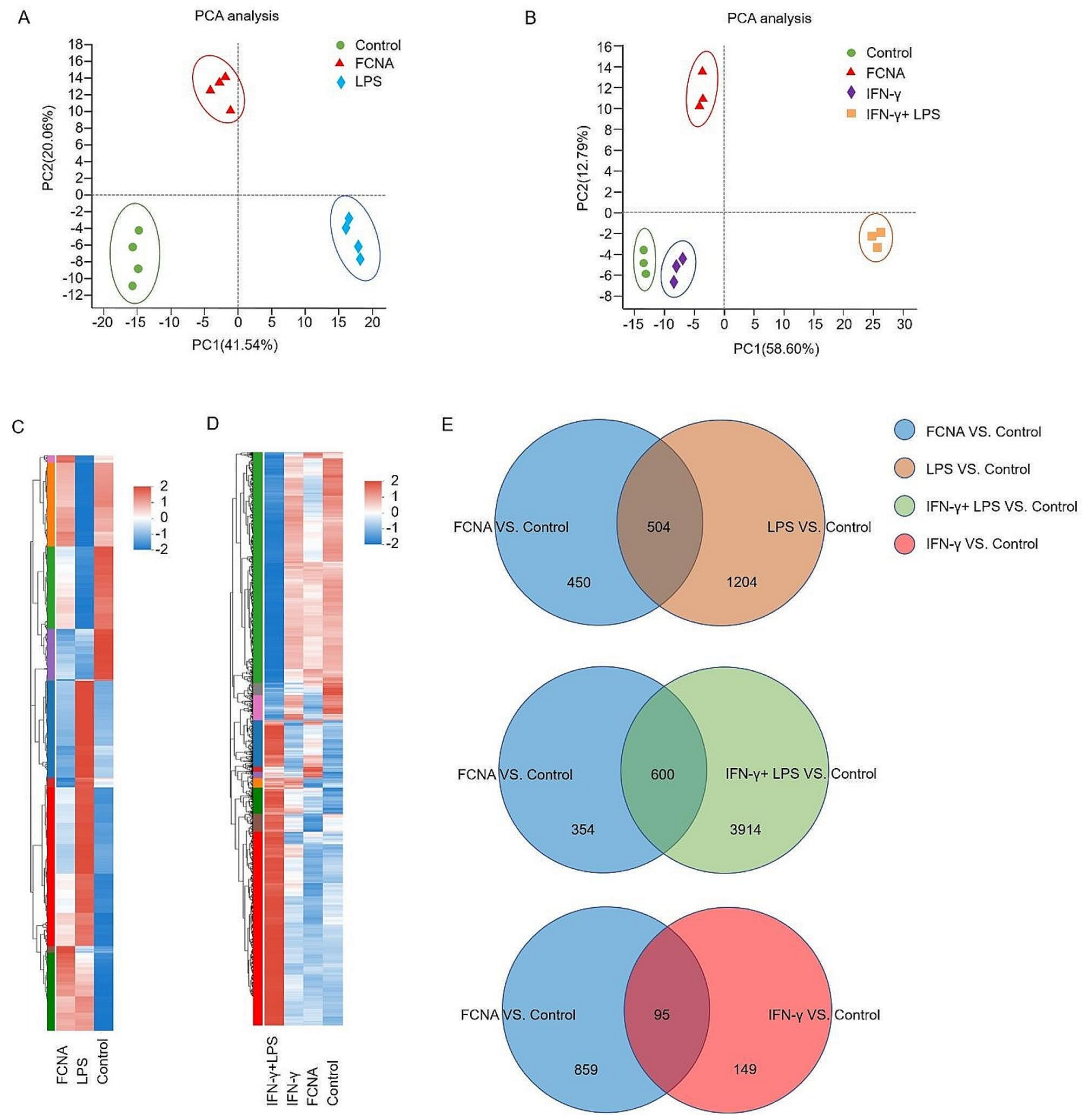
FCNA activates a distinct transcriptional program in macrophages. **A** Principal-component analysis of murine BMDMs stimulated with FCNA or LPS showing the divergence of transcriptomic programs. **B** Principal-component analysis of BMDMs stimulated with FCNA, IFN-γ or IFN-γ + LPS showing the differences in transcriptional program. **C** and **D** Clusted heatmap of normalized differentially expressed genes (DEGs) of BMDMs treated with FCNA, LPS, IFN-γ or IFN-γ + LPS. **E**. Venn diagram showing the overlap between genes upregulated in macrophages treated with FCNA, LPS, IFN-γ or IFN-γ + LPS

To address the genome-wide contribution of FCNA in non-primed macrophages, RNA sequencing (RNA-seq) was performed on murine BMDMs stimulated with FCNA, LPS, IFN-γ and IFN-γ + LPS. Principal-component analysis (PCA) shows the divergence of BMDMs treated with FCNA or LPS (Fig. 1A). Moreover, another PCA plot shows four separated clusters: Control, FCNA, IFN-γ and IFN-γ + LPS, which indicates the divergence of transcriptional program of FCNA treated macrophages compared with those stimulated with IFN-γ and IFN-γ + LPS (Fig. 1B). Hierarchical clustering plot demonstrates that a significant transcriptomic shift between control, FCNA and LPS treated BMDMs, suggesting a distinct macrophage profile activated by FCNA (Fig. 1C). Simultaneously, FCNA contributes to a different transcriptomic pattern compared with IFN-γ and IFN-γ + LPS on macrophage, as shown in another Hierarchical clustered heatmap plot (Fig. 1D). Next, we found that 53% (504/954) of DEGs in FCNA versus Control group and 30% (504/1708) of DEGs in LPS versus Control group were similarly upregulated. While 47% (450/954) of DEGs were uniquely upregulated by FCNA compared to LPS. Meanwhile, according to our data, 37% (354/954) of DEGs were exclusively upregulated by FCNA when compared to IFN-γ + LPS. 90% (859/954) of DEGs were upregulated by FCNA compared to IFN-γ (Fig. 1E). Above all, our data showed that FCNA activated a different transcriptomic profile compared to LPS, IFN-γ and IFN-γ + LPS in macrophages.

### FCNA activates the inflammatory genes in macrophages

**Fig. 2:**
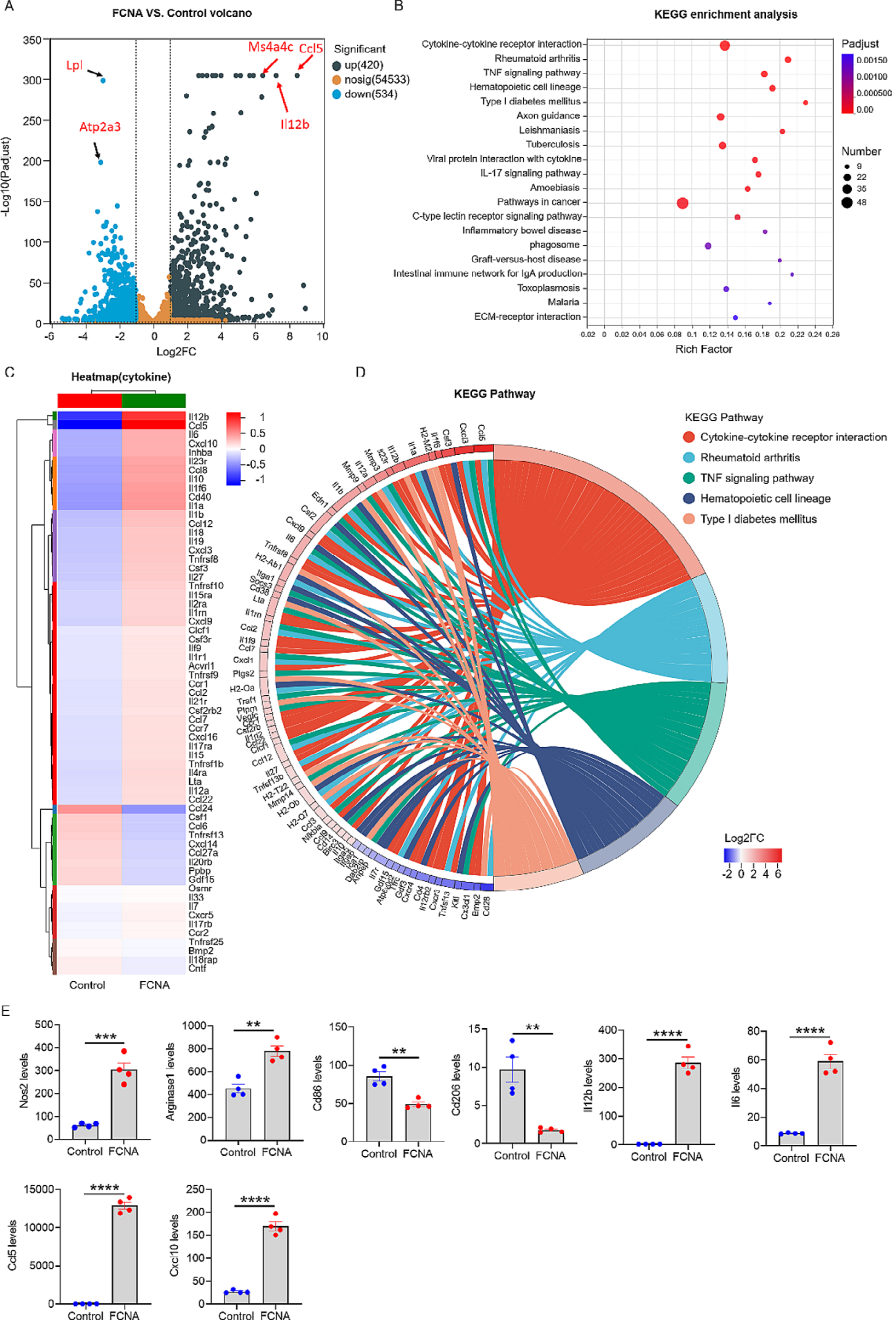
Inflammatory genes were activated in BMDMs by FCNA. **A** Volcano plot of gene expressions in FCNA-treated BMDMs. The grey dots and blue dots indicate upregulated and downregulated DEGs, respectively. **B** KEGG gene set analysis showing biological signaling pathways that are differentially activated by FCNA. **C** Heatmap shows DEGs enriched in Cytokine-cytokine receptor interaction pathway. **D** The KEGG enrichment chord diagram was constructed to visualize ranked DEGs corresponding to KEGG terms. **E** Selected gene expression levels examined by RNA-Seq analysis. Each dot represents an individual cell sample. Error bars show the mean ± SEM. *, *P* < 0.05; **, *P* < 0.01; ***, *P* < 0.001; ****, *P* < 0.0001

We have previously demonstrated that FCNA activated macrophage to a phenotype distinct from classical phenotypes induced by LPS, IFN-γ and IFN-γ + LPS. Our data further indicated that FCNA promoted macrophage polarization towards a pro-inflammatory phenotype. Volcano plot shows that 420 genes were upregulated and 534 genes were downregulated by FCNA. Specifically, genes such as Ccl5, Il12b and Ms4a4c were significantly upregulated, while Lpl and Atp2a3 were downregulated in macrophages (Fig. 2A). To explore the gene signatures of FCNA-treated macrophages, we conducted KEGG enrichment analysis. KEGG enrichment analysis identified the top-ranked biological processes “Cytokine-cytokine receptor interaction”, “Rheumatoid arthritis”,” TNF signaling pathway”, all of which are linked to inflammatory functional signatures (Fig. 2B). Genes related to “Cytokine-cytokine receptor interaction” were further categorized in a hierarchical clustered heatmap. The heatmap revealed 50 genes upregulated by FCNA, most of which were pro-inflammatory genes including Il12b, Ccl5, Il6, Cxcl10, Il1a, Il1b, Il10 and so on (Fig. 2C). Furthermore, the KEGG enrichment chord diagram depicted the corresponding relationships between the ranked upregulated DEGs and top five KEGG pathways (Fig. 2D). Consistently, most of the top-ranked upregulated genes were associated with the aforementioned top three inflammatory KEGG pathways, strongly suggesting that FCNA activated macrophage into a pro-inflammatory phenotype. Next, representative genes expression levels, which was previously recognized as markers for M1 and M2 macrophages, were evaluated by RNA-Seq. We found that FCNA upregulated Nos2, Arginase 1, Il12b, Il6, Ccl5, Cxcl10, while downregulated Cd86 and Cd206 in macrophages (Fig. 2E). Collectively, these data revealed the pro-inflammatory feature of FCNA-treated macrophages and suggested they might be an individual phenotype distinct from the classical M1 and M2.

### FCNA activates macrophage to a phenotype different from classical phenotypes

**Fig. 3:**
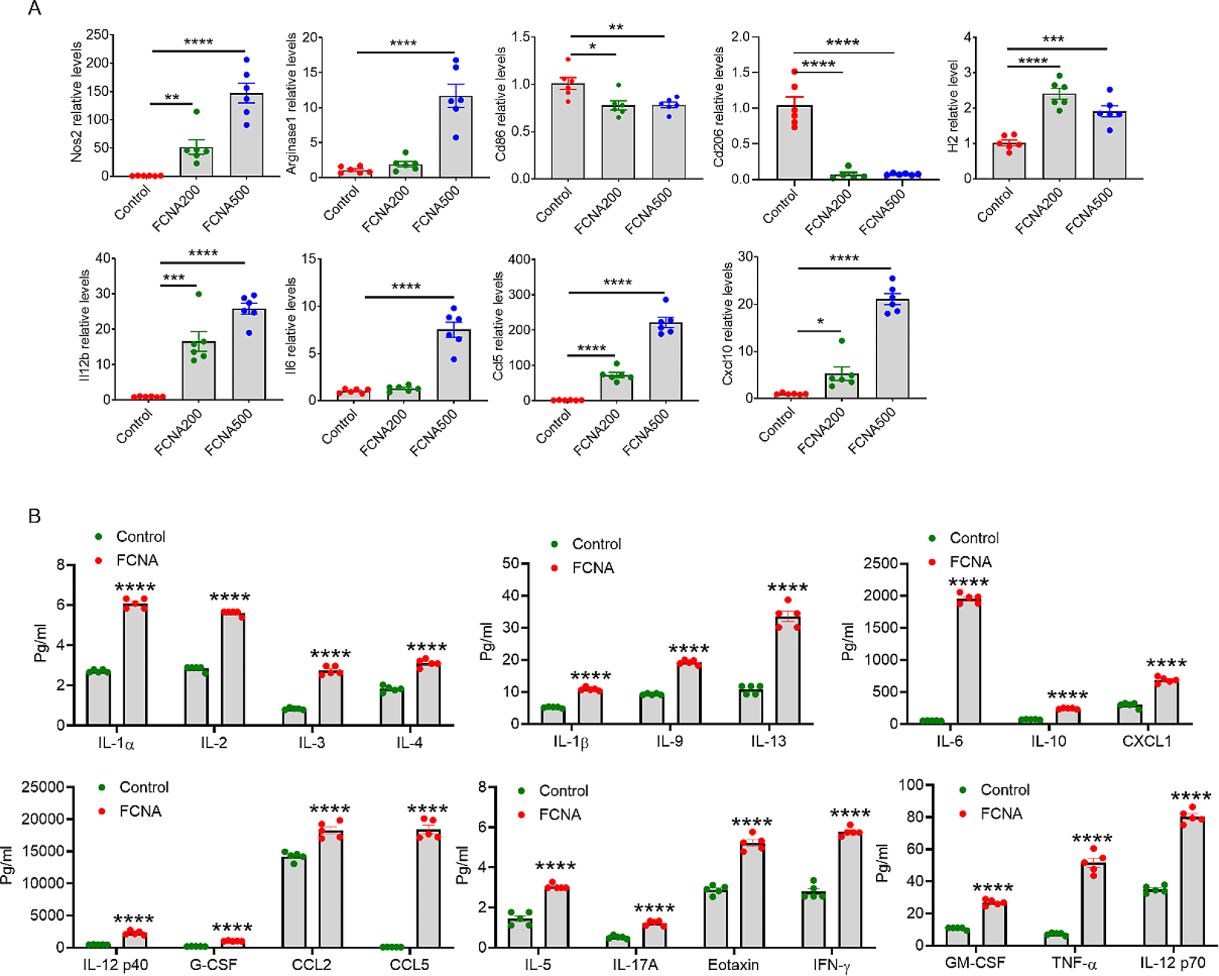
Validation of featured genes identified by RNA-Seq. **A** qPCR was performed to validate representative gene expressions regulated by FCNA (200ng/ml, 500ng/ml) in BMDMs. **B** Analysis of Luminex assay showing the secretion of cytokines in supernatants of FCNA-treated BMDMs. Error bars show the mean ± SEM. *, *P* < 0.05; **, *P* < 0.01; ***, *P* < 0.001; ****, *P* < 0.0001

Expression levels of classical macrophage markers, including Nos2, Arginase1, Cd86, Cd206 and H2 were detected by qPCR in BMDMs treated with 200ng/ml and 500ng/ml FCNA to confirm our earlier findings. We found that M1-type macrophage signature maker Nos2 and M2-type macrophage signature maker Arginase1 were both upregulated by FCNA in a dose dependent manner. While Cd86 and Cd206, which were respectively identified as classical M1- and M2-type macrophage signature markers, were both downregulated by FCNA. In addition, the antigen presenting gene H2 was activated by FCNA indicating the activation of macrophages. To validate the RNA-Seq data, qPCR was also performed on a set of selected genes top-ranked in the “Cytokine-cytokine receptor interaction” KEGG enrichment analysis pathways. According to our data, Il12b, Ccl5, Il6 and Cxcl10 were markedly upregulated by FCNA in a dose dependent manner, consistent with our RNA-Seq analysis (Fig. 3A).

To further identify the pro-inflammatory function of FCNA on macrophages, Luminex assay was performed on cell culture supernatants from BMDMs treated with or without FCNA (500 ng/ml). In line with our RNA-Seq findings, IL12 p40, IL12 p70, CCL5 and IL6 protein levels were significantly upregulated in the cell culture supernatants of FCNA group compared to control. Moreover, interleukins including IL1α, IL1β, IL2, IL3, IL4, IL5, IL9, IL10, IL13 and IL17A were significantly elevated in FCNA-treated macrophages supernatants. Chemokines including CXCL1, CCL2, CCL5 and Eotaxin were found in higher concentrations after FCNA treatment. Other inflammatory cytokines including G-CSF, GM-CSF, IFN-γ and TNF-α were also released at higher levels in FCNA group compared to control group (Fig. 3B).

### FCNA promotes macrophage polarization through activating inflammatory signaling pathways

**Fig. 4:**
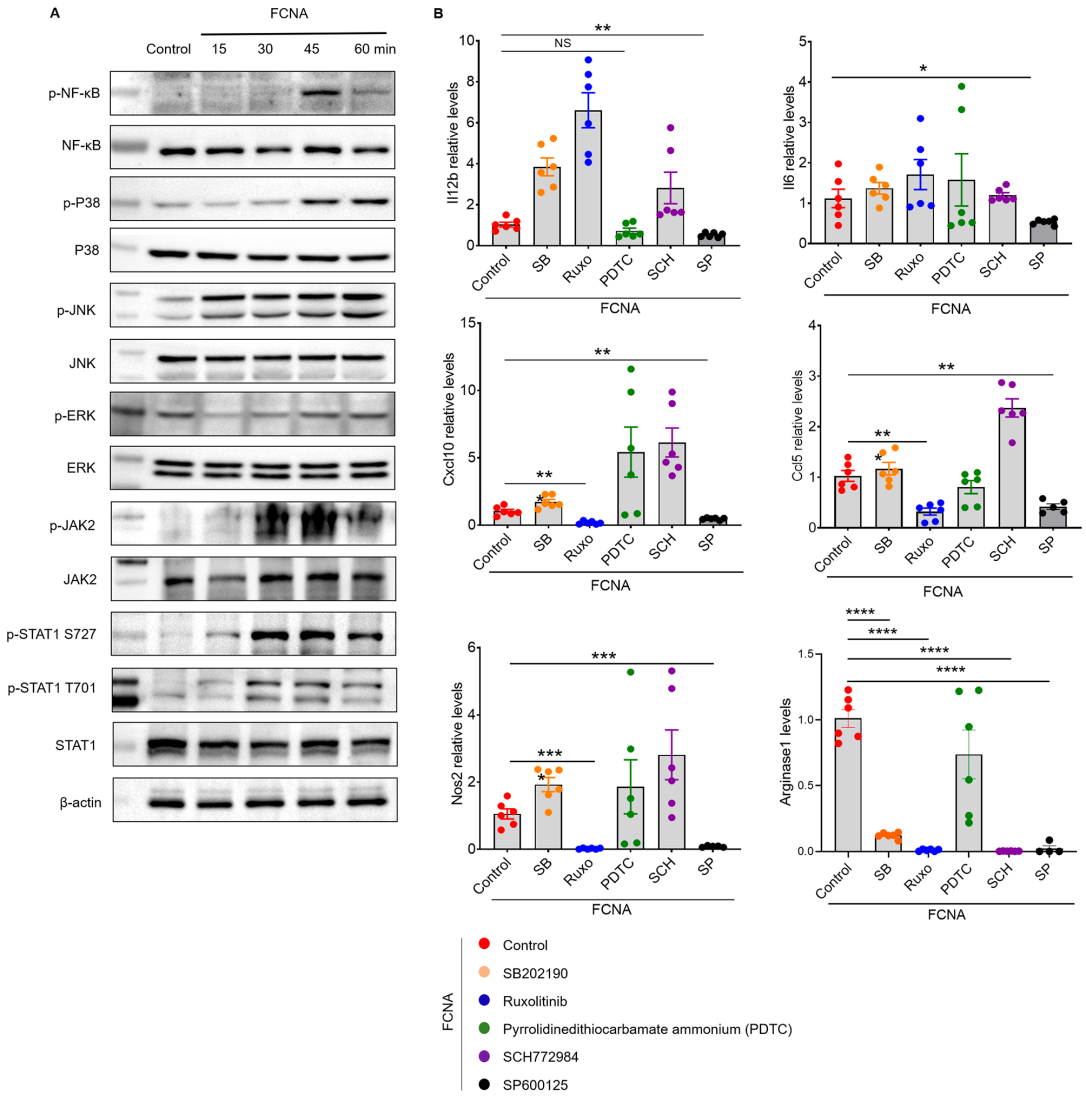
FCNA activated inflammatory signaling pathways in macrophages. **A** Protein analysis by western blot in BMDMs treated with FCNA at various timepoints. **B** Representative genes expressions were assessed by qPCR in FCNA-treated BMDMs following signaling inhibitor treatments, including p38 inhibitor (SB202190), JAK-STAT inhibitor (Ruxolitinib), NF-κb inhibitor (Pyrrolidinedithiocarbamate ammonium, PDTC), ERK inhibitor (SCH772984), and JNK inhibitor (SP600125). Data are representative of three independent experiments. Error bars show the mean ± SEM. *, *P* < 0.05; **, *P* < 0.01; ***, *P* < 0.001; ****, *P* < 0.0001

Previous studies have reported that various cell signaling pathways are activated through the process of macrophage polarization [26–28]. Here, we examined the key signaling pathways that drive macrophage activation to explore the effects of FCNA on signaling transduction. Western blot showed that FCNA promoted the activation of NF-κb signal, even though the level of phosphor-NF-κb slightly decreased after the stimulation was extended to 60 min. Activation of MAPKs, including ERK, Jun N-terminal protein kinase (JNK), and p38, was also critical for proinflammatory cytokine expression during macrophage activation. In macrophages stimulated by FCNA, we found that p38 and JNK signalings were activated significantly while ERK signaling was inhibited (Fig. 4A). IFN-γ-mediated JAK-STAT1 signaling pathway was associated with increased NOS2, MHC-II, and IL-12 expression in macrophages. The expression of NOS2, MHC-II, and IL-12 were early found upregulated by FCNA. Thus, we further examined whether JAK-STAT1 signaling pathway was activated by FCNA. In line with our assumption, FCNA triggered JAK-STAT1-mediated tyrosine phosphorylation on STAT1, indicating the activation of JAK-STAT1 signaling [29] (Fig. 4A).

Next, to further confirm the activation of these different signals, the inhibitors targeting different cell signaling pathways were administrated to BMDMs under the stimulation of FCNA. We have discovered earlier that FCNA-stimulated macrophages exhibit a high capacity to express IL-12b, IL-6, CCL5, CXCL10, NOS2 and Aginase-1, which differs from classical M1 and M2 type macrophages. We then assessed the expression of these genes under the stimulation of FCNA and various signaling inhibitors. As expected, the expression of IL-6 and IL-12b were inhibited by SP600125, a JNK signaling pathway inhibitor (Fig. 4B). CCL5, CXCL10 and NOS2 were decreased by both JAK-STAT1 signaling pathway inhibitor Ruxolitinib and JNK pathway inhibitor SP600125 (Fig. 4B). However, even though the Pyrrolidinedithiocarbamate ammonium (PDTC) and SB202190 (targeting NF-κb and p38 respectively) have not significantly reduced the expression of the target genes we examined, there might be other genes regulated by these pathways. Consistent with our western blot results, SCH772984, the inhibitor of ERK signaling pathway, has no capacity changing the target genes expressions (Fig. 4B). Notably, it is controversial that all inhibitors except for PDTC, inhibited the expression of Arginase1 by FCNA. Further studies are required to elucidate the underlying mechanisms. Altogether, our data showed that FCNA activated macrophage polarization through activating the inflammatory signaling pathways, including NF-κb, p38, JNK and JAK-STAT1.

### FCNA deteriorates rheumatoid arthritis symptoms

**Fig. 5:**
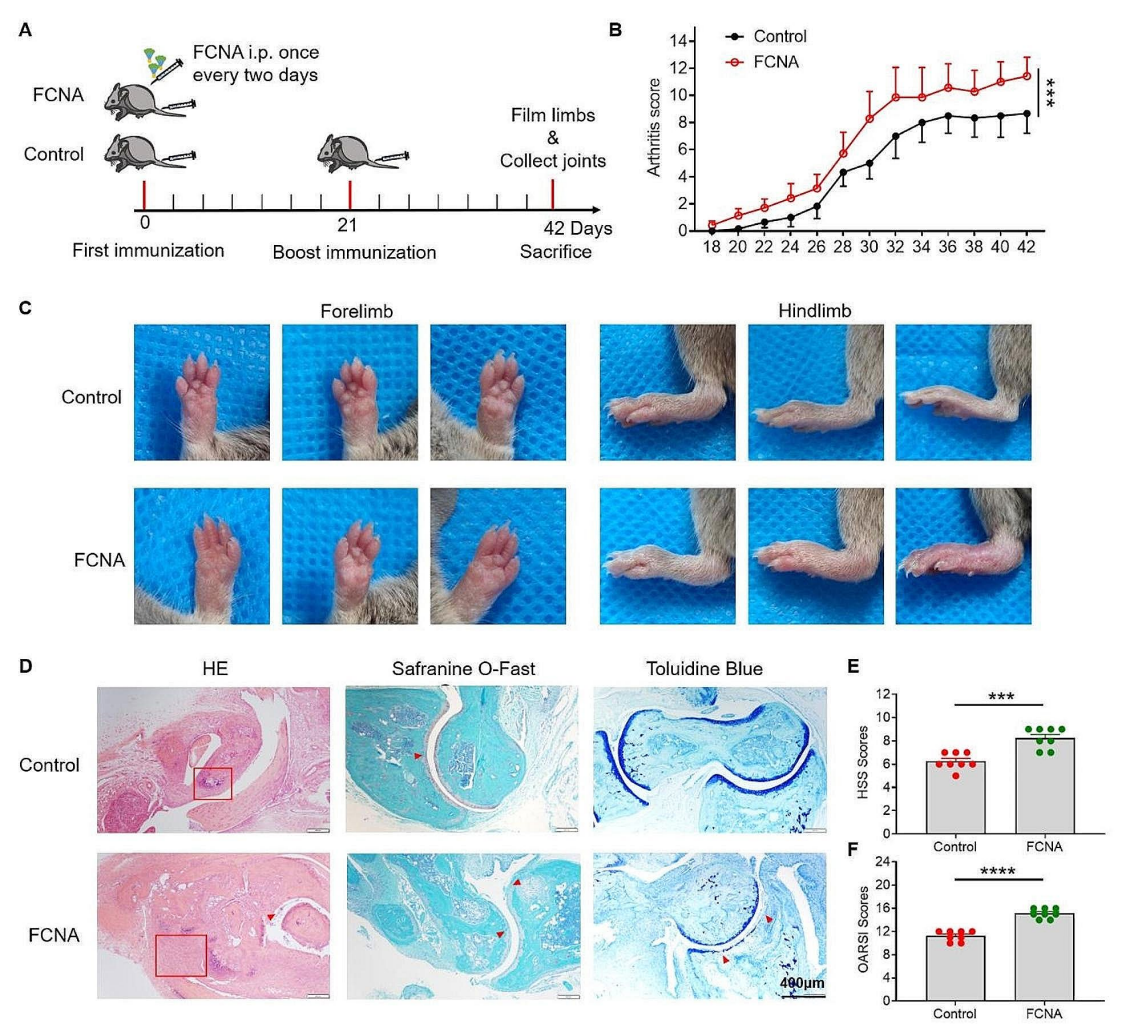
FCNA treatment deteriorates CIA symptoms. **A** DBA/1 mice were immunized on Day0 and 21 to induce the CIA model, with the treatment of FCNA (5 µg/mouse) i.p. once every two days. **B** Arthritis scores are measured every two days (Control, *n* = 8; FCNA, *n* = 8). **C** Representative images of forelimbs and hindlimbs from different mice on Day42 post immunization. **D** The morphology of articular cartilage and synovial invasion were observed by H&E staining, safranine O-fast green staining and toluidine blue staining of mice ankle joints. Scale bars, 400 μm. **E** and **F** Quantification of H&E staining by HSS scores and OARSI scores (*n* = 8/group). The P value was determined by two-way ANOVA (**B**) and unpaired Student’s t test (**E, F**). Error bars show the mean ± SEM. *, *P* < 0.05; **, *P* < 0.01; ***, *P* < 0.001; ****, *P* < 0.0001

Previous studies have confirmed the essential role of macrophages in the progression of rheumatoid arthritis (RA) [30–32]. Our RNA-Seq analysis, aligned with KEGG pathway data, substantiates that FCNA induces the activation of macrophages toward a distinctive pro-inflammatory phenotype, potentially contributing to the pathogenesis of RA. To examine the impact of FCNA on macrophages in the context of RA, we employed the collagen-induced arthritis (CIA) model. Mice were injected with FCNA intraperitoneally every two days just after the first immunization. The severity of arthritis was assessed by daily observation initiated 15 days after the first immunization (Fig. 5A). We found that FCNA group showed a higher arthritis clinical score compared to control group throughout the experimental period, indicating that FCNA exacerbates the inflammatory response associated with RA (Fig. 5B). In accordance with the clinical score, administration of FCNA resulted in severe redness and swelling in the paws. Furthermore, we observed a significant increase in paw thickness in FCNA-treated group compared to control group (Fig. 5C). Histopathological evaluation of ankle joints sections, based on hematoxylin and eosin (H&E) staining, revealed severe synovial lining cell layer hypertrophy and hyperplasia accompanied by pronounced inflammatory infiltration in FCNA-treated group relative to the control group. Additionally, the FCNA-treated group exhibited marked activation of synovial stroma, characterized by a dense distribution of fibroblasts and endothelial cells (Fig. 5D). Histological Synovitis Score (HSS) and the Modified Osteoarthritis Research Society International (OARSI) score were evaluated to manifested the degree of inflammatory synovial tissue infiltration and the severity of articular cartilage damage based on H&E staining (Fig. 5E and F). Safranine O-fast green staining and toluidine blue staining showed severe cartilage erosion and damage in FCNA group than control group (Fig. 5D). Taken together, our findings suggest that FCNA aggravates the inflammation and damage in CIA mice, thereby aggravating RA symptoms.

### Deletion of FCNA attenuates rheumatoid arthritis symptoms

**Fig. 6:**
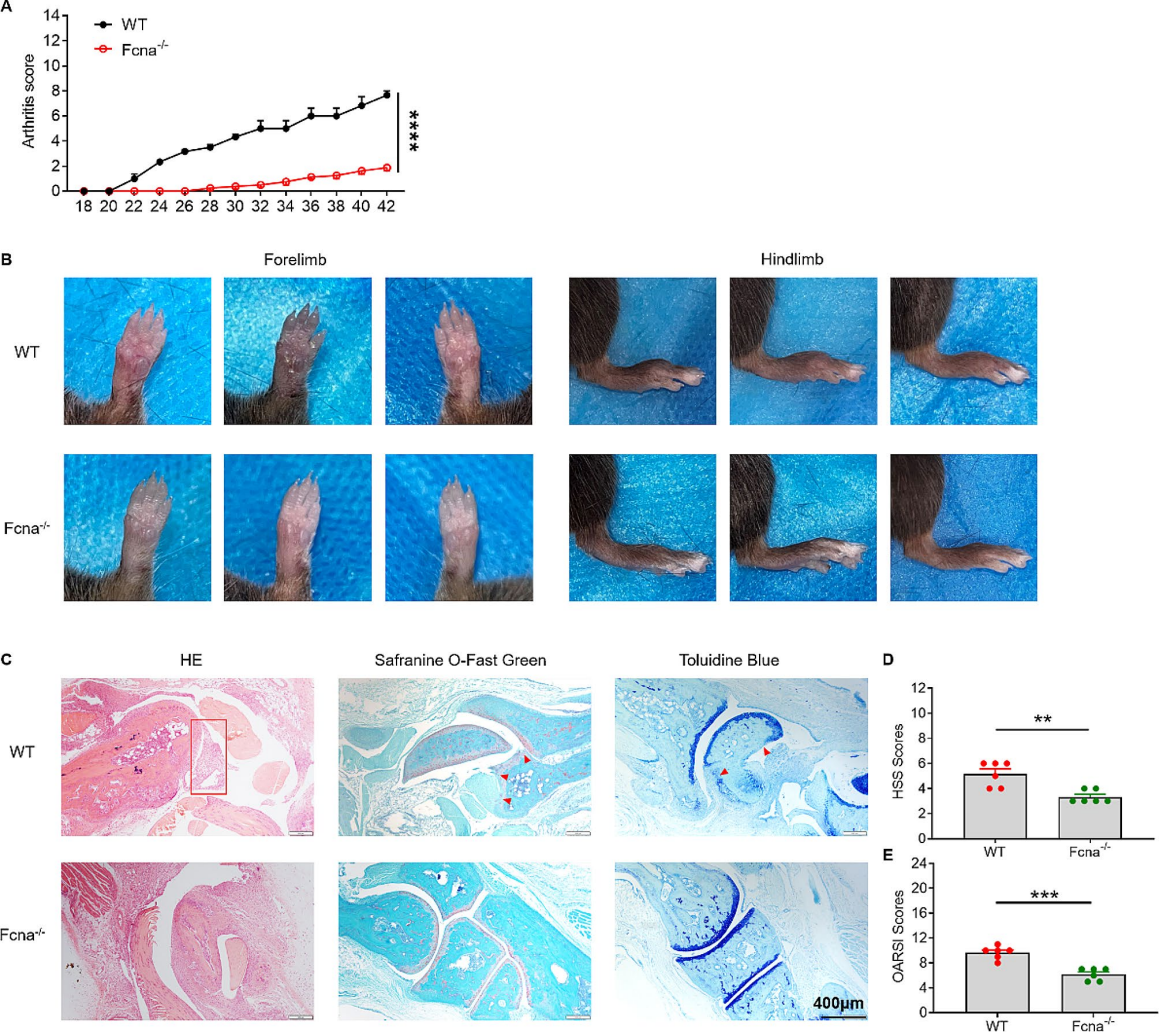
Deletion of FCNA attenuates CIA symptoms. **A** Arthritis scores are measured every two days (WT, *n* = 6; Fcna-/-, *n* = 8) after the induction of CIA model. **B** Representative images of forelimbs and hindlimbs from different mice on Day42 post immunization. **C** The morphology of articular cartilage and synovial invasion were observed by H&E staining, safranine O-fast green staining and toluidine blue staining of mice ankle joints. Scale bars, 400 μm. **D** and **E** Quantification of H&E staining by HSS scores and OARSI scores (*n* = 6/group). The P value was determined by two-way ANOVA and unpaired Student’s t test. Error bars show the mean ± SEM. *, *P* < 0.05; **, *P* < 0.01; ***, *P* < 0.001; ****, *P* < 0.0001

To further explore the role of FCNA in Rheumatoid arthritis pathogenesis, the FCNA knock out mice were generated (Supplementary Fig. 1). Both Fcna^−/−^mice and WT littermate controls were immunized with collagen. The clinical signs of CIA were scored on a daily basis. WT mice exhibited markedly more severe clinical signs throughout the course compared to Fcna^−/−^mice (Fig. 6A). Paw thickness further confirmed the attenuation of redness and swelling in both forelimb and hindlimb paws of Fcna^−/−^mice (Fig. 6B). Histopathological analysis based on H&E, Safranine O-Fast Green and Toluidine Blue staining revealed significant reduction of synovial inflammation, pannus formation, and cartilage and bone destruction in Fcna^−/−^ mice joints (Fig. 6C). Consistent with these observations, HSS and OARSI scores indicated diminished inflammatory synovial tissue infiltration and articular cartilage damage in Fcna^−/−^mice (Fig. 6D and E). In accordance with our assumption, deletion of FCNA attenuates the symptoms of RA.

### Deletion of FCNA ameliorates pathogenesis of DSS-induced colitis

**Fig. 7:**
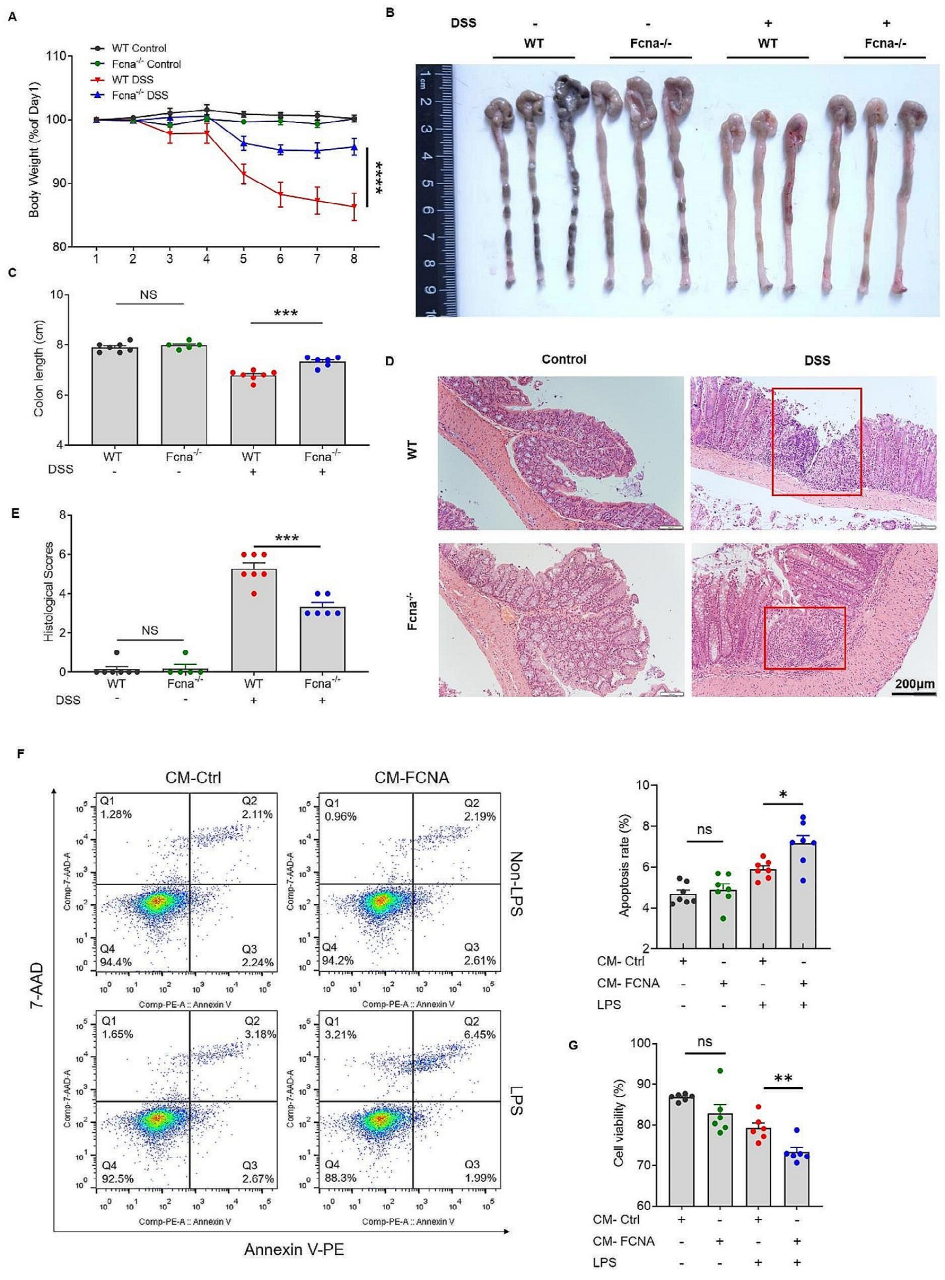
FCNA deletion ameliorates DSS-induced colitis. **A** Body weight changes of each experimental group during DSS treatment (WT control, *n* = 7; Fcna^−/−^ Control, *n* = 5; WT DSS, *n* = 7; Fcna^−/−^ DSS, *n* = 6). **B** Representative images of colon tissues from each group (*n* = 3/group). **C** Bar graph showing the analysis of colon lengths in each group. **D** Histological analysis of colon tissues showed by H&E staining. Scale bars, 200 μm. **E** Histological scores of the colon damages were evaluated. **F, G** MODE-K cells were cultured with conditioned medium (CM) from Control or FCNA-treated BMDMs, and stimulated with or without LPS. The cells were examined for apoptosis by flow cytometry (**F**), and cell viability by CCK-8 (**G**). The P value was determined by two-way ANOVA and unpaired Student’s t test. Error bars show the mean ± SEM. *, *P* < 0.05; **, *P* < 0.01; ***, *P* < 0.001; ****, *P* < 0.0001

DSS-induced colitis has been considered to be driven by activation of intestinal macrophages which cause tissue damage and infiltration of neutrophils and dendritic cells through secreting pro-inflammatory cytokines and chemokines in the colon [33]. To better understand the role of FCNA in macrophage polarization, we conducted the DSS-induced colitis mouse model characterized by acute colonic tissue inflammation in Fcna^−/−^ and WT littermate mice. We first recorded the body weight variation between age- and sex-matched Fcna^−/−^ and WT mice without oral administration of DSS in drinking water, showing no differences between the two groups. However, WT mice suffered a more significant body weight loss than Fcna^−/−^ mice upon given DSS from day one (Fig. 7A). Colon length, a parameter with low variability in the DSS-induced colitis model, was also measured to gauge the severity of colitis [34]. On average, the colons of WT mice were shorter than Fcna^−/−^mice after DSS adminstration, which indicated an adverse effect of FCNA in DSS model (Fig. 7B and C). Representative colon sections were prepared to determine the histopathological changes. In line with the clinical signs of colitis, severe transmural inflammation with focal areas of extensive ulceration and necrotic lesions were observed in WT group in contrast with Fcna^−/−^group. While there was no evidence of histological damage in the WT and Fcna^−/−^ group without DSS administration (Fig. 7D). A semiquantitative analysis of these histological parameters confirmed greater histopathological changes in DSS-fed WT mice compared to Fcna^−/−^mice (Fig. 7E). To further elucidate the role of FCNA-stimulated macrophages in colitis, we conducted in vitro experiments on LPS absent or stimulated MODE-K cells. MODE-K cells were cultured with conditioned medium (CM) of Control or FCNA-stimulated BMDMs. In LPS-stimulated MODE-K cells cultured in CM-FCNA, cell apoptosis was elevated (Fig. 7F), and cell viability was significantly reduced (Fig. 7G). Taken together, our data demonstrated the important role of FCNA in the inflammatory process of DSS challenge.

**Fig. 8:**
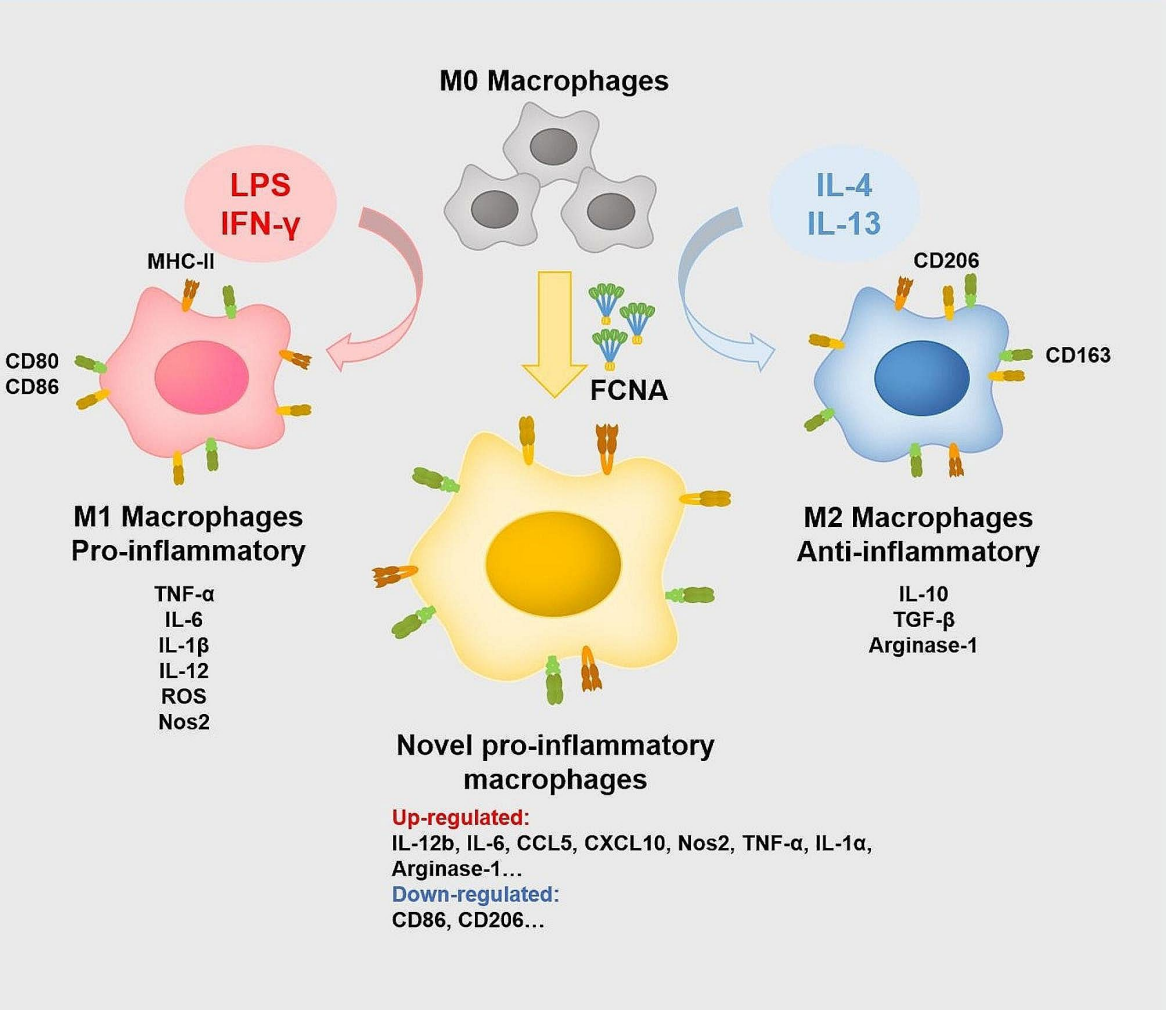
Graphical summary of FCNA activates macrophages into a distinct phenotype. FCNA activates M0 type macrophage polarizing into a novel pro-inflammatory phenotype. This distinctive phenotype is characterized by upregulation of interleukins including IL-12b, IL-6, IL-1a, TNF-α etc., chemokines including CCL5, CXCL10 etc., and macrophage signature markers iNOS and Arginase 1. The novel phenotype is also recognized with downregulation of CD86 and CD206

## Discussion

Macrophages play important roles in inflammatory conditions and autoimmune diseases. Since the transcriptional signatures and functional phenotypes of macrophages are regulated by triggering cues in the local environment, exploring activators affecting macrophage polarization is essential for the further characterization of the complex biology of macrophages. Here, the investigation of genome-wide transcriptomic analysis revealed FCNA activates a distinct inflammatory phenotype of macrophages. Through the administration of exogenous FCNA or the knockdown of FCNA in mouse models of RA and IBD, we have further substantiated the role of FCNA in macrophage polarization and its contribution to the progression and pathogenesis of autoimmune diseases. Collectively, we have found that FCNA activated macrophage into a novel pro-inflammatory phenotype.

Classical pro-inflammatory macrophage phenotype, or M1 macrophages, were typically induced by LPS and IFN-γ [35]. We found that FCNA activated macrophages to a distinct phenotype with a unique gene expression profile compared to LPS/ IFN-γ-stimulated activation. Overall, genes upregulated by FCNA predominantly enriched in inflammatory processes. Expressions of pro-inflammatory cytokines and chemokines were highly elevated in FCNA-treated macrophages, with an increase in NOS2, a notable marker for pro-inflammatory M1 macrophages. While, Arginase 1 as a classical marker for anti-inflammatory M2 macrophages was also upregulated by FCNA. In addition, high expression of CD86 and CD206, which was found as signatures of M1 and M2 respectively, were both downregulated by FCNA. All these controversial data challenge the categorization of this new phenotype as M1 or M2 macrophages, which encourages us to define a novel phenotype of macrophages characterized by high expression of pro-inflammatory cytokines, chemokines, iNOS, Arginase 1, and low expression of CD86 and CD206. It should be noted that Yang YF. et al. have reported on FCNA increasing iNOS expression while decreasing Arginase 1 expression in RAW264.7 cells [36]. The result from this study is different with our current work. RAW264.7 cells is a monocyte/macrophage-like cell line originating from Abelson leukemia virus transformed cell line derived from BALB/c mice. In our study, however, we used bone marrow-derived macrophages (BMDMs), which is the primary cell type of macrophages. Certain differences between BMDMs and RAW264.7 cells have been demonstrated by previous studies [37–39]. Therefore, the differences in Arginase 1 expression may come from the different cell types used for in vitro studies or the variations of experimental conditions.

To validate the RNA-Seq findings, a selected set of genes were quantified by qPCR. In general, a good correspondence was found between the RNA-seq and qPCR results. Pro-inflammatory cytokines including Il12b and Il6, and chemokines including Ccl5 and Cxcl10, were found increased by FCNA. Signatures of M1 and M2 macrophages including Nos2 and Arginase 1 were both upregulated, while other signatures such as Cd86 and Cd206 were downregulated. To further confirm the pro-inflammatory role of FCNA on macrophages, cytokines and chemokines in cell culture supernatant were detected by Luminex assay. All of the pro-inflammatory cytokines and chemokines were found increased by FCNA, however, some anti-inflammatory cytokines including IL-4, IL-10 were also increased by FCNA. These findings further support our point that FCNA activates macrophages to a new phenotype.

Studies showed that the activation of cell signaling pathways directs macrophage polarization. As an important inflammatory regulator, NF-κB promotes macrophages polarizing to M1 subtype and expressing pro-inflammatory cytokines [40]. P38 MAPK, which is a pivotal pathway subject to MAPKs, has been proved to regulate the activation of M1 macrophages [41–44]. Other MAPKs pathways including JNK and ERK also played roles in promoting macrophage activation and polarization [41–43]. Moreover, the IFN-γ-mediated macrophage polarization toward an “M1-like” state is mediated by the activation of JAK-STAT1 signaling pathways [45, 46]. All of the above signaling pathways are involved in macrophage polarization, and have been detected in the present study, where NF-κB, P38, JNK and JAK-STAT1 pathways were activated by FCNA in macrophages. With the administration of inhibitors specific for different signaling pathways and the evaluation of signatured gene expressions, we further confirmed the activation of these pathways by FCNA, which provide mechanistic clarification on the effects of FCNA on macrophages.

The balance of M1/M2 macrophages determines the severity and progression of inflammatory and autoimmune diseases [47, 48]. In the pathogenesis of RA, certain factors activate macrophage polarization to inflammatory phenotypes that deteriorate the pathophysiological outcomes and result in a bad prognosis [49]. On the contrary, recent advances have proved that the shift of macrophages to an anti-inflammatory subtype could ameliorate the symptoms of RA [50, 51]. Thus, exploring factors regulating macrophage polarization provides insights for the development of new therapeutic treatments for RA. A clinical study has reported higher FCN2 concentrations in the serum of RA patients and a positive correlation between disease severity and FCN2 level [12]. Therefore, we investigated the impact of FCNA on macrophages in vivo by administrating FCNA to CIA model mice via intraperitoneal injections, and we further validated these findings by inducing CIA in Fcna^−/−^ mice. In line with the in vitro findings, FCNA regulated the macrophage polarization to a novel pro-inflammatory phenotype and deteriorated the symptoms and pathological damages. Compelling evidence has proved that macrophages are the central drivers of IBD [52–54]. Especially, clinical remission in IBD is associated with reduced pro-inflammatory macrophage activity [55, 56]. We then investigated the effect of FCNA on macrophages through constructing the DSS-induced IBD model. Our results showed that knocking down Fcna ameliorated the severity of clinical symptoms and pathology of DSS model mice. Based on the central role of macrophages in autoimmune diseases, we assumed that FCNA may activate a new pro-inflammatory subtype of macrophage to aggravate the diseases. However, studies reported that FCNA activated the lectin pathway through forming complex with MASP-2 [57]. In this study, we have not ruled out the role of lectin pathway on macrophage differentiation which was activated by FCNA. We still need to investigate whether the effect of FCNA on macrophages is independent of the activation of complement cascade.

Taken together, our data identified a novel pro-inflammatory macrophage phenotype activated by FCNA. This subtype of macrophages aggravates the symptoms and pathological damages of autoimmune diseases such as RA and IBD. Our findings indicate a promising therapeutic approach for a wide range of diseases associated with dysregulated macrophage activation. Despite the exciting findings we have evidenced, it still calls for further explorations in human on ficolins and the novel phenotype of macrophages.

There are a few limitations in this study: (1) Additional experiments using human recombinant ficolin-2 on human original macrophages will be helpful confirming the role of Ficolin-2 on macrophage polarization and supporting the results from this study. (2) As Ficolin-A are identified as innate soluble pattern recognition molecules that form complexes with MASP-2 to activate the complement cascade, further study needs to be conducted to investigate the role of Ficolin-A/ MASP-2 complexes on macrophages. (3) Ficolin-B, which shares similarities with Ficolin-A, should be used in further study to investigate its role on macrophage differentiation.

### Electronic supplementary material

Below is the link to the electronic supplementary material.

Supplementary Material 1

## Data Availability

No datasets were generated or analysed during the current study.
