# Supplementary material for: Ficolin-A induces macrophage polarization to a novel pro-inflammatory phenotype distinct from classical M1
Source: Cell Commun Signal. 2024 May 15;22:271. doi: 10.1186/s12964-024-01571-4 (PMC11094856; doi:10.1186/s12964-024-01571-4)

|  | **Forward** | **Reverse** |
| --- | --- | --- |
| **iNOS** | GTTCTCAGCCCAACAATACAAGA | GTGGACGGGTCGATGTCAC |
| **Arginase1** | GGTCTTAGGCTCAGGCCATTA | CGCTTATGGTGGAAACCTCTC |
| **CD86** | TTACGGAAGCACCCACGATG | ACTACCAGCTCACTCAGGCT |
| **IL-6** | GACAAAGCCAGAGTCCTTCAGAGAG | CTAGGTTTGCCGAGTAGATCTC |
| **IL-12b** | TGGTTTGCCATCGTTTTGCTG | ACAGGTGAGGTTCACTGTTTCT |
| **CCL5** | TGCCCACGTCAAGGAGTATTT | CCCACTTCTTCTCTGGGTTGG |
| **CXCL10** | CCAAGTGCTGCCGTCATTTTC | GGCTCGCAGGGATGATTTCAA |
| **CD206** | CTCTGTTCAGCTATTGGACGC | CGGAATTTCTGGGATTCAGCTTC |
| **CXCL9** | AAACCTGCCTAGATCCGGAC | CGACTTTGGGGTGTTTTGGG |
| **MCH-II** | GTGTGAGTCCTGGTGACTGC | CACTCGCCCATGAACTGGTA |
| **β-actin** | AGATGTGGATCAGCAAGCAG | GCGCAAGTTAGGTTTTGTCA |

**Table S1** The sequences of RT-qPCR primers.

| **Product Name** | **Item No.** | **Company** |  |
| --- | --- | --- | --- |
| Dulbecco’s Modified Eagle Medium | | 10566016 | Invitrogen |
| Fetal bovine serum | | 10099158 | Gibco |
| Lipopolysaccharide | | L5293 | Sigma-Aldrich |
| Recombinant Mouse IFN-gamma Protein | | 485-MI | R&D Systems |
| Recombinant Mouse Ficolin-1 (Fcn1) | | CSB-EP008550MOa0 | CUSABIO |
| SB202190 | | S1077 | Selleck |
| Ruxolitinib (INCB018424)  Pyrrolidinedithiocarbamate ammonium  SCH772984  SP600125 | | S1378  S3633  S7101  S1460 | Selleck  Selleck  Selleck  Selleck |
| Phospho-NFκB mAb  NFκB mAb  Phospho-IκBα mAb  IκB Antibody  Phospho-p38 MAPK mAb  p38 MAPK mAb  Phospho-p44/42 MAPK (Erk1/2) mAb  p44/42 MAPK (Erk1/2) mAb  Phospho-SAPK/JNK mAb  SAPK/JNK (E7R5D) mAb  Phospho-Jak2 mAb  Jak2 (D2E12) mAb  Phospho-STAT1 (Ser727)  Phospho-STAT1 (Tyr701)  STAT1 mAb  β-actin mAb  Immunization Grade Bovine Type II Collagen | | 3033s  8242S  2859S  9242S  4511S  8690S  4370  4695  4668  67096  3776  9258  8826S  7649S  9172S  4970  20022 | Cell Signaling Technology  Cell Signaling Technology  Cell Signaling Technology  Cell Signaling Technology  Cell Signaling Technology  Cell Signaling Technology  Cell Signaling Technology  Cell Signaling Technology  Cell Signaling Technology  Cell Signaling Technology  Cell Signaling Technology  Cell Signaling Technology  Cell Signaling Technology  Cell Signaling Technology  Cell Signaling Technology  Cell Signaling Technology  Chondrex |
| Freund’s Adjuvant, Complete | | F5881 | Sigma-Aldrich |
| Dextran Sulfate Sodium Salt | | 02160110-CF | MP Biomedicals |

**Table S2** Reagents and Antibodies used in the research.

| **Feauture** | **Score** |
| --- | --- |
| **Hyperplasia or enlargement of synovial lining cell layer** |  |
| Absent | 0 |
| Slight enlargement (2-3 cell layers). Giant cells are scarce | 1 |
| Moderate enlargement (4-5 cell layers). Some giant cells or lymphocytes | 2 |
| Strong enlargement (≥6 cell layers). Giant cells and lymphocytes are frequent | 3 |
| **Inflammatory infiltration** |  |
| Absent | 1 |
| Slight inflammatory infiltration | 1 |
| Moderate inflammatory infiltration | 2 |
| Strong inflammatory infiltration | 3 |
| **Activation of synovial stroma/pannus formation** |  |
| Absent | 0- |
| Slight synovial stroma activation | 1 |
| Moderate synovial stroma activation | 2 |
| Strong synovial stroma activation | 3 |

**Table S3** The scoring scheme of Histological Synovitis Score (HSS).

| **Feature** | **Score** |
| --- | --- |
| **Structure** |  |
| Normal | 0 |
| Slight surface irregularities | 1 |
| Moderate surface irregularities | 2 |
| Severe surface irregularities | 3 |
| Clefts/fissures into transitional zone (1/3 depth) | 4 |
| Clefts/fissures into radial zone (2/3 depth) | 5 |
| Clefts/fissures into calcified zone (full depth) | 6 |
| Fibrillation and/or erosion to transitional zone (1/3 depth) | 7 |
| Fibrillation and/or erosion to radial zone (2/3 depth) | 8 |
| Fibrillation and/or erosion to calcified zone (full depth) | 9 |
| Fibrillation and/or erosion to subchondral bone | 10 |
| **Cellularity** |  |
| Normal | 0 |
| Increase or slight decrease | 1 |
| Moderate decrease | 2 |
| No cells present | 3 |
| **Chondrocyte cloning** |  |
| Normal | 0 |
| Several doublets | 1 |
| Many doublets | 2 |
| Doublets and triplets | 3 |
| Multiple cell nests | 4 |

**Table S4** The scoring scheme of Modified Osteoarthritis Research Society International

(OARSI) scores.

| **Inflammatory cell infiltrate** |  |  |
| --- | --- | --- |
| **Severity** | **Extent** | **Score** |
| Mild | Mucosa | 1 |
| Moderate | Mucosa and submucosa | 2 |
| Marked | Transmural | 3 |
| **Intestinal architecture** |  |  |
| **Epithelial changes** | **Mucosal architecture** | **Score** |
| Focal erosions |  | 1 |
| Erosions | ±Focal ulcerations | 2 |
|  | Extended ulcerations ± granulation tissue ± pseudopolyps | 3 |
| **Sum** |  | **0-6** |

**Table S5** The scoring scheme of histological scores for DSS-induced colitis mouse model.

**Figure S1** Comparison of the sizes of different organs from WT and Fcna^-/-^ mice (8 weeks-old), including spleens, lymphoid nodes, kidneys, hearts, and colons.


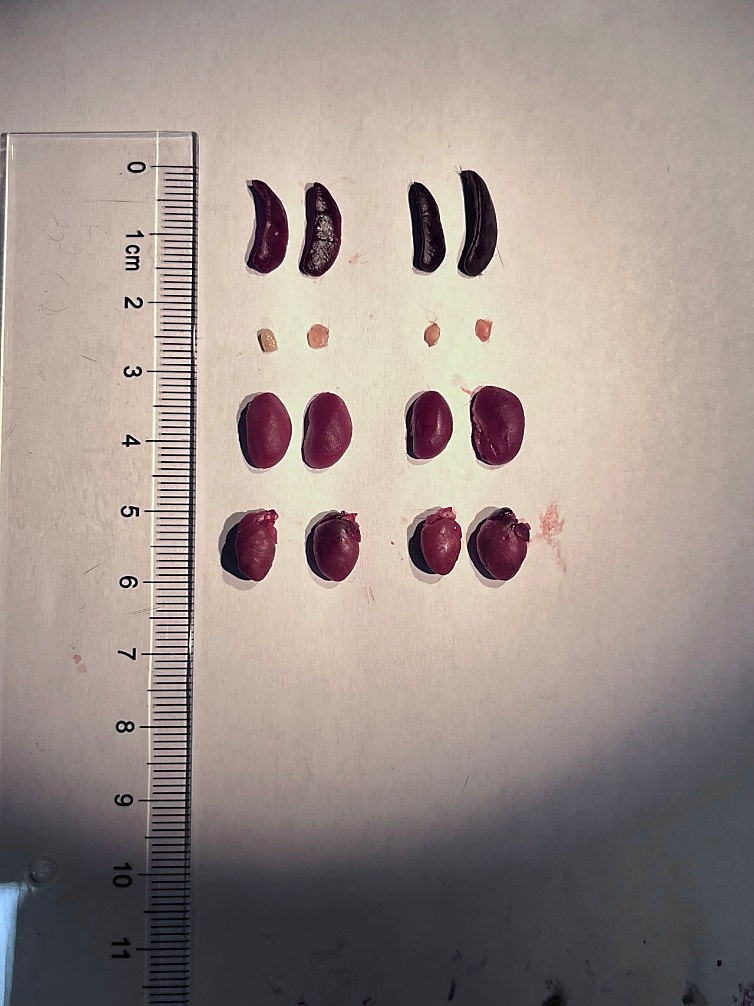

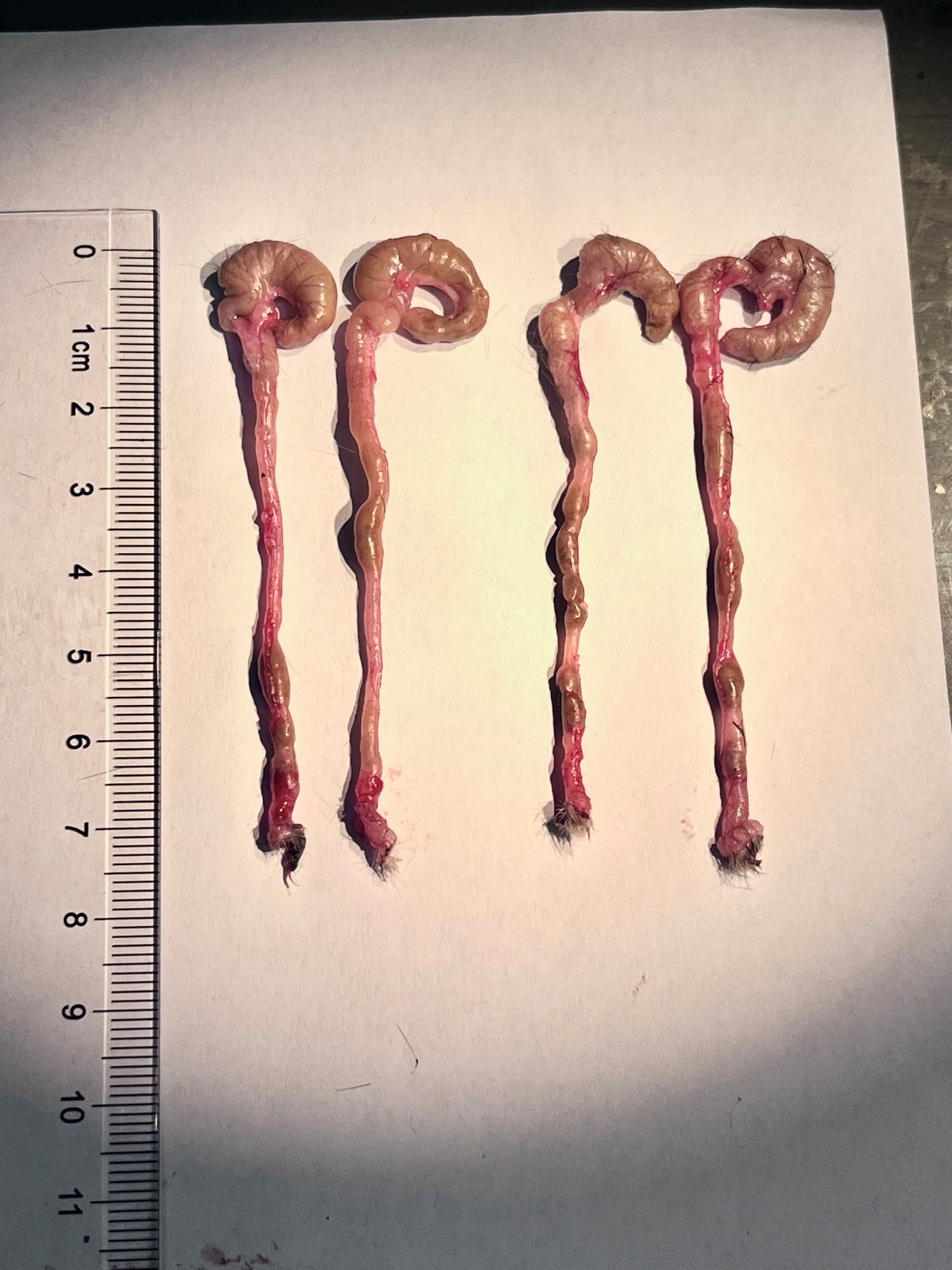


WT

WT

Fcna-/-

Fcna-/-

All full unprocessed original images of gels and western blots related to **Fig. 4**:

1.
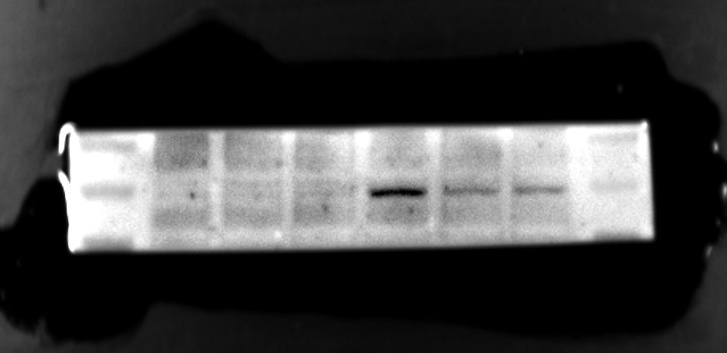
p-NF-κB (65 kDa)
2. NF-κB (65 kDa)


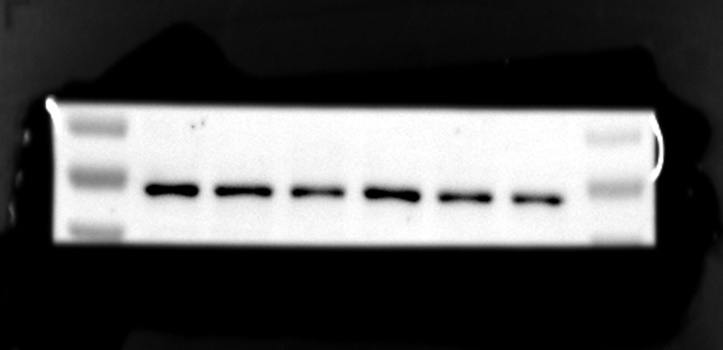


1.
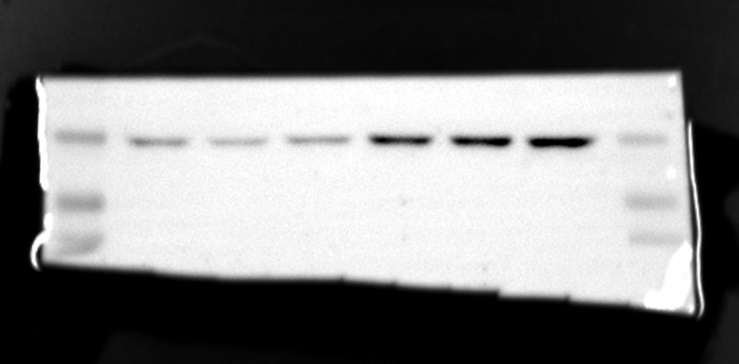
p-P38 (43 kDa)
2.
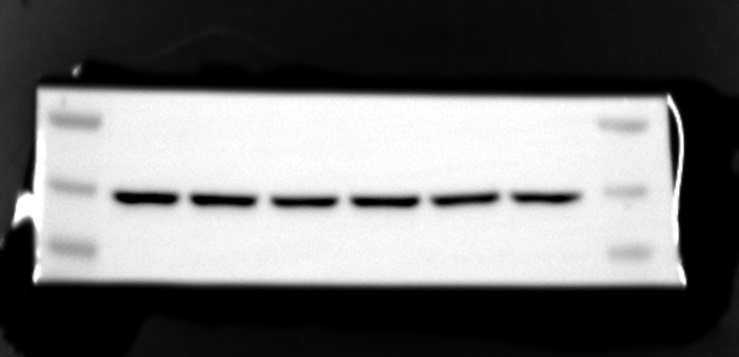
P38 (40 kDa)
3. p-JNK (46, 54 kDa)


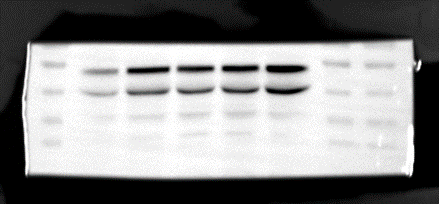


1. JNK (46, 54 kDa)


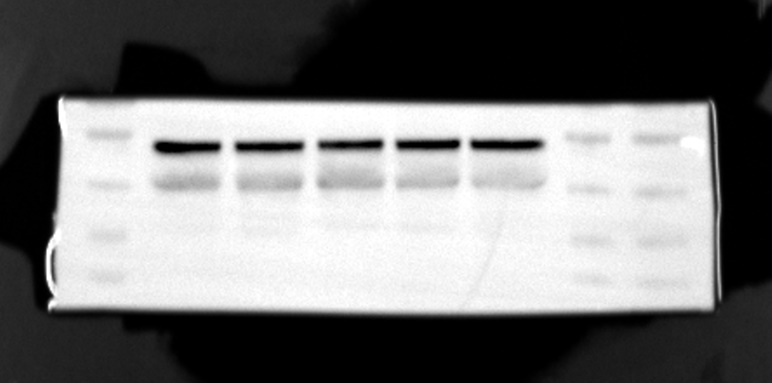


1.
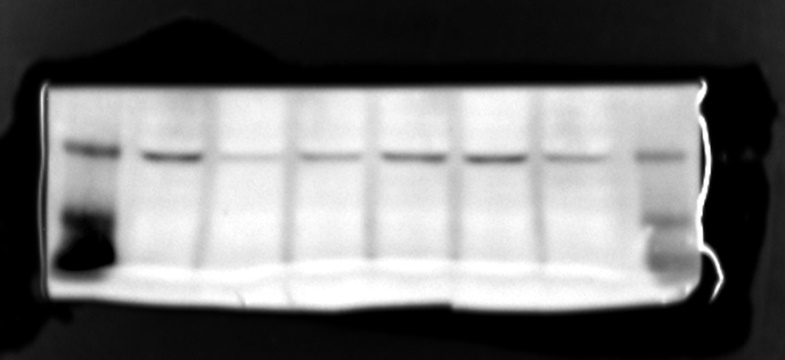
p-ERK (42, 44 kDa)
2.
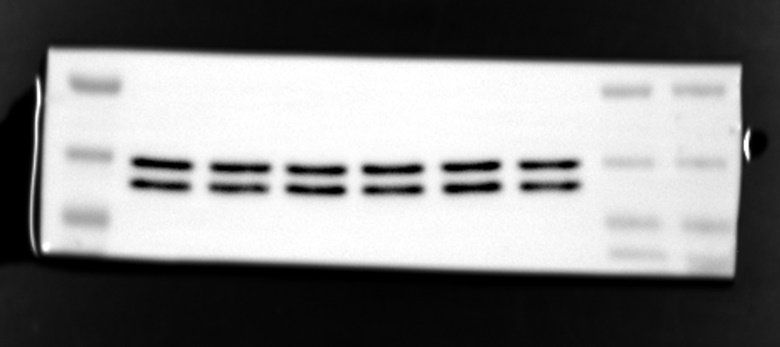
ERK (42, 44 kDa)
3.
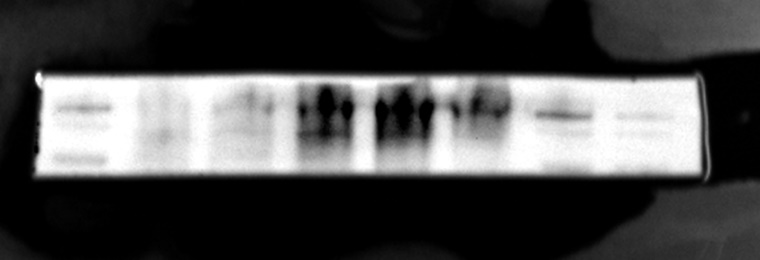
p-JAK2 (125 kDa)
4.

JAK2 (125 kDa)
5.
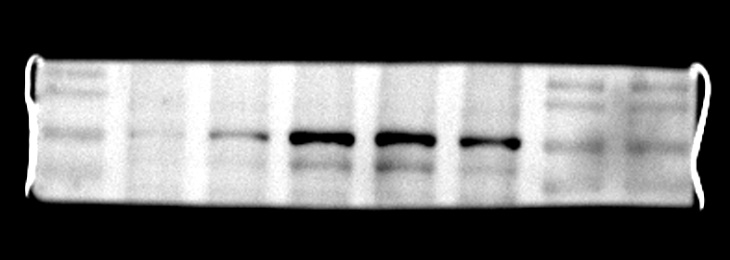
p-STAT1 S727 (91 kDa)
6.
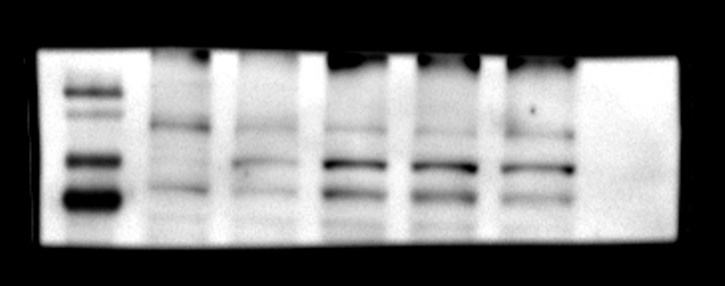
p-STAT1 T701 (84, 91 kDa)
7. STAT1 (84, 91 kDa)


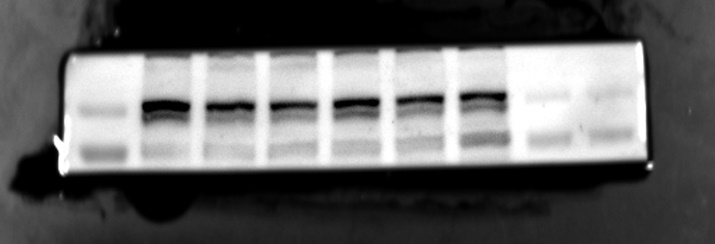


1. β-actin (45 kDa)


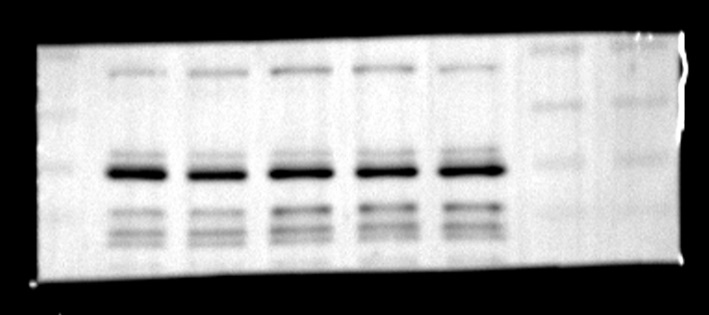

Supplement: Supplementary file 1 — Supplementary Material 1 [file 12964_2024_1571_MOESM1_ESM.docx]
